# *In vitro* and *in vivo* antileishmanial activity of β-acetyl-digitoxin, a cardenolide of *Digitalis lanata* potentially useful to treat visceral leishmaniasis

**DOI:** 10.1051/parasite/2021036

**Published:** 2021-04-14

**Authors:** Camila S. Freitas, Daniela P. Lage, João A. Oliveira-da-Silva, Rafaella R. Costa, Débora V.C. Mendonça, Vívian T. Martins, Thiago A.R. Reis, Luciana M.R. Antinarelli, Amanda S. Machado, Grasiele S.V. Tavares, Fernanda F. Ramos, Rory C.F. Brito, Fernanda Ludolf, Miguel A. Chávez-Fumagalli, Bruno M. Roatt, Gabriela S. Ramos, Jennifer Munkert, Flaviano M. Ottoni, Priscilla R.V. Campana, Mariana C. Duarte, Denise U. Gonçalves, Elaine S. Coimbra, Fernão C. Braga, Rodrigo M. Pádua, Eduardo A.F. Coelho

**Affiliations:** 1 Programa de Pós-Graduação em Ciências da Saúde: Infectologia e Medicina Tropical, Faculdade de Medicina, Universidade Federal de Minas Gerais Belo Horizonte 30130-100 Minas Gerais Brazil; 2 Departamento de Parasitologia, Microbiologia e Imunologia, Instituto de Ciências Biológicas, Universidade Federal de Juiz de Fora Juiz de Fora 36036-900 Minas Gerais Brazil; 3 Laboratório de Imunopatologia, Núcleo de Pesquisas em Ciências Biológicas, Departamento de Ciências Biológicas, Insituto de Ciências Exatas e Biológicas, Universidade Federal de Ouro Preto Ouro Preto 35400-000 Minas Gerais Brazil; 4 Universidad Católica de Santa María Urb. San José S/N Umacollo 04000 Arequipa Perú; 5 Departamento de Produtos Farmacêuticos, Faculdade de Farmácia, Universidade Federal de Minas Gerais Belo Horizonte 31270-901 Minas Gerais Brazil; 6 Departament Biologie, LS Pharmazeutische Biologie, Universität Erlangen-Nürnberg 91054 Erlangen Germany; 7 Departamento de Patologia Clínica, COLTEC, Universidade Federal de Minas Gerais Belo Horizonte 31270-901 Minas Gerais Brazil

**Keywords:** Treatment, β-acetyl-digitoxin, Visceral leishmaniasis, Drug repositioning, Toxicity, Miltefosine

## Abstract

Current treatments of visceral leishmaniasis face limitations due to drug side effects and/or high cost, along with the emergence of parasite resistance. Novel and low-cost antileishmanial agents are therefore required. We report herein the antileishmanial activity of β-acetyl-digitoxin (b-AD), a cardenolide isolated from *Digitalis lanata* leaves, assayed *in vitro* and *in vivo* against *Leishmania infantum*. Results showed direct action of b-AD against parasites, as well as efficacy for the treatment of *Leishmania*-infected macrophages. *In vivo* experiments using b-AD-containing Pluronic^®^ F127 polymeric micelles (b-AD/Mic) to treat *L. infantum*-infected mice showed that this composition reduced the parasite load in distinct organs in more significant levels. It also induced the development of anti-parasite Th1-type immunity, attested by high levels of IFN-γ, IL-12, TNF-α, GM-CSF, nitrite and specific IgG2a antibodies, in addition to low IL-4 and IL-10 contents, along with higher IFN-γ-producing CD4^+^ and CD8^+^ T-cell frequency. Furthermore, low toxicity was found in the organs of the treated animals. Comparing the therapeutic effect between the treatments, b-AD/Mic was the most effective in protecting animals against infection, when compared to the other groups including miltefosine used as a drug control. Data found 15 days after treatment were similar to those obtained one day post-therapy. In conclusion, the results obtained suggest that b-AD/Mic is a promising antileishmanial agent and deserves further studies to investigate its potential to treat visceral leishmaniasis.

## Abstract (fr)

Les traitements actuels de la leishmaniose viscérale font face à des limitations dues aux effets secondaires des médicaments et/ou à leur coût élevé, ainsi qu’à l’émergence d’une résistance parasitaire. Des agents antileishmaniaux nouveaux et peu coûteux sont donc nécessaires. Nous rapportons ici l’activité antileishmaniale de la β-acétyl-digitoxine (b-AD), un cardénolide isolé à partir de feuilles de *Digitalis lanata*, mesurée *in vitro* et *in vivo* contre *Leishmania infantum*. Les résultats ont montré une action directe de la b-AD contre les parasites, ainsi qu’une efficacité pour le traitement des macrophages infectés par *Leishmania*. Des expériences *in vivo* utilisant des micelles polymériques Pluronic^®^ F127 contenant de la b-AD (b-AD/Mic) pour traiter des souris infectées par *L. infantum* ont montré que cette composition réduisait à des niveaux plus significatifs la charge parasitaire dans différents organes, ainsi que le développement d’une immunité antiparasitaire de type Th1, attestée par les taux élevés d’IFN-γ, d’IL-12, de TNF-α, de GM-CSF, de nitrite et d’anticorps IgG2a spécifiques, en plus des faibles taux d’IL-4 et IL-10, ainsi qu’une fréquence plus élevée des cellules T CD4^+^ and CD8^+^ productrices d’ IFN-γ. De plus, une faible toxicité a été trouvée dans les organes des animaux traités. En comparant l’effet thérapeutique des traitements, b-AD/Mic était le plus efficace pour protéger les animaux contre l’infection, par rapport aux autres groupes comprenant la miltefosine utilisée comme contrôle médicamenteux. Les données trouvées 15 jours après le traitement étaient similaires à celles obtenues un jour après le traitement. En conclusion, les résultats obtenus suggèrent que b-AD/Mic est un agent antileishmanial prometteur et mérite des études supplémentaires pour étudier son potentiel à traiter la leishmaniose viscérale.

## Introduction

Leishmaniases are vector-borne diseases caused by distinct species of protozoan parasites of the genus *Leishmania*. This disease complex is endemic in several countries in the world, where approximately 380 million people are at risk and 2.0 million new cases are registered per year [67]. Leishmaniases have distinct clinical manifestations, including tegumentary leishmaniasis (TL), which involves the cutaneous, mucosal and diffuse-cutaneous clinical forms, and visceral leishmaniasis (VL), which can be fatal if acute and untreated [33]. Disease control methods are not effective; the current treatment of human cases causes toxicity and/or has a high cost, in addition to the emergence of resistant parasite strains [62].

Visceral leishmaniasis is caused by *Leishmania infantum* in Latin America, Central Asia, and the Mediterranean region [3]. In symptomatic disease, splenomegaly, fever, anemia, weight loss, and weakness are commonly observed in the patients [66]. Ideally, a suitable treatment should be safe, non-toxic, and effective against parasites. Since the late 1940s, treatment is based on the use of pentavalent antimonials; however, these compounds are toxic, require intravenous or intramuscular administration, which is uncomfortable for the patients, and parasite resistance has increased [13, 49]. Amphotericin B (AmpB) has also been used as a treatment option. Although effective against *Leishmania* parasites, its toxicity is high. Lipid formulations have reduced AmpB toxicity and shown high efficacy; however, the high cost is still an impeditive factor [44]. Miltefosine has been also used as a therapeutic option in several countries, and it was the first oral drug administered against human VL. However, miltefosine causes teratogenicity and parasite resistance has been also registered [19]. Within this context, there is a need to research and develop novel and low-cost antileishmanial agents.

Drug discovery is a long and expensive process. In this regard, drug repositioning could be considered and tests using compounds with other known biological applications could be evaluated as antileishmanial agents [4, 6, 10]. Cardenolides are glycosides clinically used for over 200 years, with the mechanism of action based on the inhibition of Na^+^/K^+^-ATPase, involved in the Na^+^/K^+^ pump mechanism dependent on these ions [8, 41, 46]. Cardenolides have been used for the treatment of congestive heart failure [26], and present antitumor [56], anti-inflammatory [29], antimalarial [14], anti-oxidant, and anti-aging [68] activities.

The chemical investigation of *Digitalis* species resulted in the isolation of over 80 cardenolides, ascribed as the main bioactive constituents of the genus [21, 27]. Aiming to further explore new antileishmanial candidates, in the present work, the *in vitro* and *in vivo* activity of β-acetyl-digitoxin (b-AD) cardenolide, which was isolated from the leaves of *D. lanata*, was evaluated against *L. infantum* species. *In vitro* assays showed that b-AD was effective against *L. infantum* promastigotes and amastigotes, and had low toxicity in murine and human cells. Preliminary data showed that this cardenolide derivative acts on parasite mitochondria, causing cell death. Additionally, *in vivo* treatment performed in *L. infantum*-infected mice showed that both free b-AD and a composition formed by the molecule incorporated in polymeric micelles (b-AD/Mic) resulted in significant reductions in the parasite load in the spleen, liver, bone marrow (BM) and draining lymph nodes (dLN) of the animals, when analyses were performed 1 and 15 days post-treatment. Results obtained using miltefosine were similar to those found using the free molecule, but lower as compared to those found using b-AD/Mic. In addition, anti-parasite Th1-type immunity was observed in the treated and infected animals, suggesting the possibility of testing this compound as a therapeutic target against VL.

## Materials and methods

### Drugs and chemicals

**Figure 1:**
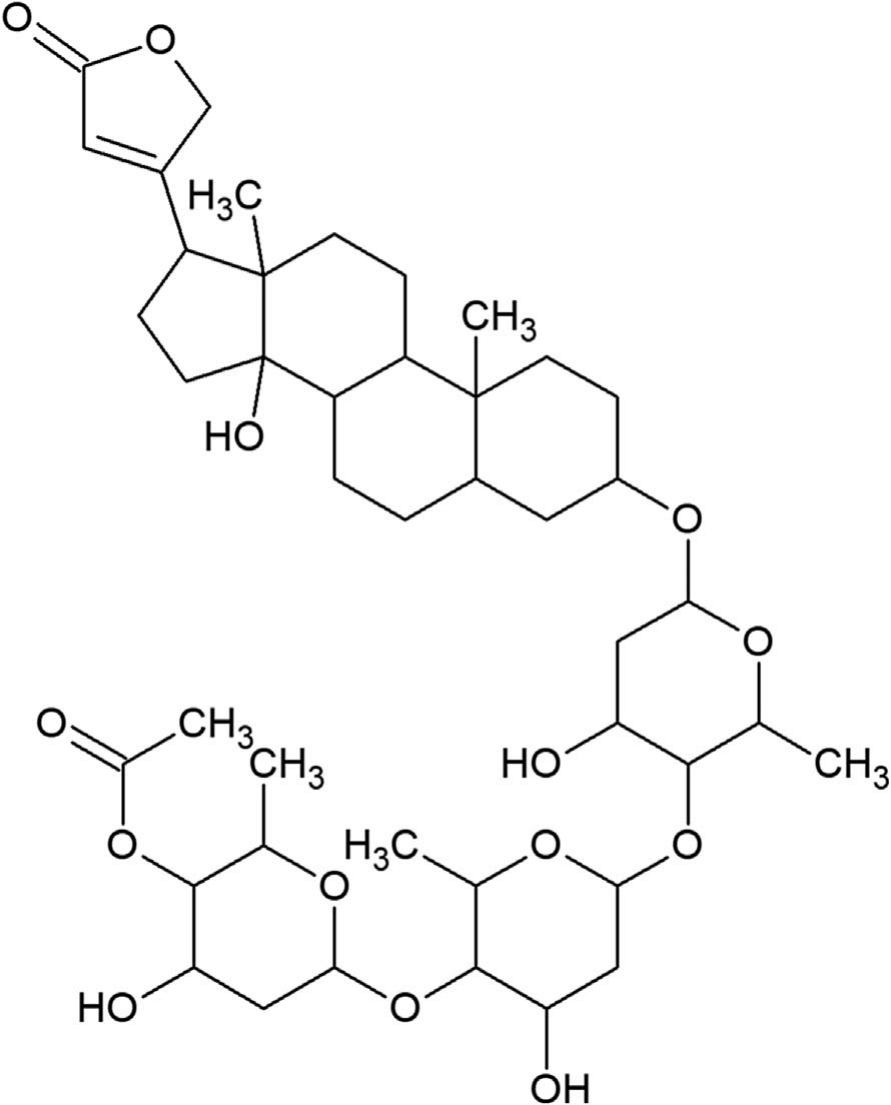
Chemical structure of β-acetyl-digitoxin.

Miltefosine, AmpB, and Poloxamer 407 (Pluronic^®^ F127) were commercially acquired and have catalog numbers 58066-85-6, 1397-89-3, and 16758, respectively (Sigma–Aldrich, St. Louis, MO, USA). β-acetyl-digitoxin (C_43_H_66_O_14_; molecular weight 807 g/mol) was extracted and purified from leaves of *Digitalis lanata* species. After isolation and purification, the structural elucidation of the compound was carried out using ultraviolet spectrophotometry, mass spectrometry, and nuclear magnetic resonance. Data obtained were analyzed and the chemical structure was clarified (Fig. 1).

### Ethics statement, experimental animals, and parasites

The work was submitted to and approved by the Ethics Committee in Animal Research (CEUA) from Federal University of Minas Gerais (UFMG; Belo Horizonte, Minas Gerais, Brazil), with protocol number 085/2017. Female BALB/c mice (6–8 weeks old) were acquired from the Institute of Biological Sciences (ICB) of UFMG and were kept under pathogen-free conditions. *Leishmania infantum* (MHOM/BR/1970/BH46) was grown in Schneider’s medium (Sigma–Aldrich) added to 20% heat-inactivated fetal bovine serum (FBS; Sigma–Aldrich) and 20 mM L-glutamine at pH 7.4, 24 °C [15].

### *In vitro* antileishmanial activity

The 50% *Leishmania* inhibitory concentration (IC_50_) was evaluated *in vitro* by incubating logarithmic phase promastigotes in the presence of b-AD (0–61.96 μM) or AmpB (0–1.08 μM) in 96-well culture plates (Nunclon, Roskilde, Denmark) for 48 h at 24 °C. Cell viability was assessed by the MTT [3-(4.5-dimethylthiazol-2-yl)-2.5-diphenyl tetrazolium bromide; Sigma–Aldrich] method. The optical density (OD) values were read in a microplate spectrophotometer (Spectra Max Plus, Molecular Devices, San Jose, CA, USA), at 570 nm. Results were entered into Microsoft Excel (version 10.0) spreadsheets and IC_50_ values were calculated by sigmoidal regression of the dose-response curve [63].

### Cytotoxicity assay

Cytotoxicity was evaluated in murine macrophages and human red blood cells, in which 50% inhibition of macrophages (CC_50_) and red blood cells (RBC_50_) was calculated. To do this, macrophages were obtained by peritoneal lavage of female BALB/c mice (*n* = 5) using 5 mL cold phosphate buffered saline (PBS 1× pH 7.4). Peritoneal exudate cells were centrifuged at 1000 ×*g* for 10 min and resuspended in RPMI 1640 medium. Macrophages (5 × 10^5^ cells/mL) were then incubated with (0–123.92 μM) or AmpB (0–10.82 μM) in RPMI 1640 medium for 48 h at 37 °C in 5% CO_2_. Cell viability was assayed by the MTT method. To evaluate hemolytic activity, a 5% human red cell suspension was incubated with b-AD (0–123.92 μM) or AmpB (0–10.82 μM) for 1 h at 37 °C in 5% CO_2_. The suspension was centrifuged at 1000 ×*g* for 10 min and the lysis percentage was read in a spectrophotometer, at 570 nm. The absence or presence of hemolysis were evaluated by replacing b-AD for PBS or distilled water, respectively. Results were entered into Microsoft Excel spreadsheets and CC_50_ and RBC_50_ values were calculated using sigmoidal regression by means of dose-response curves [63].

### Treatment of infected macrophages and inhibition of infection

Stationary phase promastigotes (5 × 10^5^) were cultured in RPMI 1640 medium added to 20% FBS and 20 mM L-glutamine pH 7.4, for 24 h at 37 °C in 5% CO_2_. Parasites were then included in the cultures, at a ratio of 10 parasites per macrophage, and the incubation was developed for 48 h at 37 °C in 5% CO_2_. Free parasites were removed by washing with medium and *Leishmania*-infected macrophages were incubated with b-AD (0, 3.09, 6.19 and 12.39 μM) or AmpB (0, 0.27, 0.54 and 1.08 μM) for 48 h at 24 °C in 5% CO_2_. In another experiment, stationary phase promastigotes (5 × 10^6^ cells) were incubated with b-AD (0, 3.09, 6.19 and 12.39 μM) or AmpB (0, 0.27, 0.54 and 1.08 μM) for 4 h at 24 °C. Parasites were washed in RPMI 1640, quantified and added in culture to infect murine macrophages, at a ratio of 10 parasites per macrophage, for 48 h at 37 °C in 5% CO_2_. After cell fixation using 4% paraformaldehyde, cells were washed and stained with Giemsa, when the infection percentage, the number of amastigotes per treated macrophage, and the reduction in the infection percentage were determined by counting 200 cells, in triplicate, using an optical microscope [39].

### Evaluation of mitochondrial membrane potential

*Leishmania infantum* promastigotes (10^7^ cells) were cultured in the absence or presence of b-AD (41.93 μM, corresponding to 2× the IC_50_ value) for 24 h at 25 °C. Parasites were washed in PBS and incubated with 500 nM MitoTracker Red CM-H2XROS (Invitrogen, USA), for 30 min in the dark and at room temperature. After washing twice with PBS, cells were added to a black 96-well plate and fluorescence intensity was measured in a fluorometer (FL × 800, BioTek Instruments, Inc., Winooski, VT, USA), with excitation and emission wavelengths of 528 nm and 600 nm, respectively. Parasites treated with carbonyl cyanide-4-(trifluoromethoxy)phenylhydrazone (FCCP; 5.0 μM) for 10 min were used as a positive control, while untreated parasites were used as a negative control [25].

### Reactive oxygen species (ROS) production

*Leishmania infantum* promastigotes (10^7^ cells) were cultured in the absence or presence of b-AD (41.93 μM, corresponding to two times the IC_50_ value) for 24 h at 25 °C. After, parasites were incubated with 20 μM cell-permeant 2′,7′-dichlorodihydrofluorescein diacetate (H_2_DCFDA; Sigma-Aldrich, USA) for 30 min in the dark and at room temperature. Fluorescence intensity was measured in a spectrofluorometer (Varioskan^®^ Flash, Thermo Scientific, USA), with excitation and emission wavelengths of 485 and 528 nm, respectively. H_2_O_2_-treated parasites (4000 μM; Sigma-Aldrich, USA) were used as a positive control, while untreated parasites were used as a negative control [25].

### Preparation of b-AD-containing micelles

The b-AD-containing micelles (b-AD/Mic) were prepared as described elsewhere [54]. Briefly, Poloxamer 407 (18% w/w) was diluted in PBS under magnetic agitation for 18 h at 4 °C. Eight milligrams of b-AD were added to dichloromethane (500 μL) and solubilized using a vortex. The mixture was added to the prepared solution under vigorous agitation and in ice bath, until a viscous emulsion was obtained. The alcohol was evaporated using a rotary evaporate (Buchi, Flawil, Switzerland), and the b-AD/Mic composition was obtained as a transparent yellow gel at 22 °C. Empty micelles were prepared (18% w/w) using the protocol described above, but without addition of b-AD.

### Mice infection and treatment regimens

Mice (*n* = 12 per group) were infected subcutaneously with *L. infantum* stationary-phase promastigotes (10^7^ parasites) and, 60 days after, they were grouped and received one of the following regimens, which was administered by the subcutaneous route: saline group: mice received PBS (50 μL); empty micelles (B/Mic) group: mice received empty micelles (10 mg/kg body weight diluted in 50 μL of PBS); miltefosine group: mice received miltefosine by oral route (2 mg/kg body weight); b-AD group: mice received b-AD (5 mg/kg body weight diluted in 50 μL of PBS), and b-AD/micelle (b-AD/Mic) group: mice received b-AD-containing micelles (5 mg/kg body weight diluted in 50 μL of PBS). Treatments were performed every two days and for 10 days. One and 15 days after treatment, half of the animals of each group were euthanized, when parasitological and immunological analyses were performed.

### Evaluation of parasite load

Organ parasitism was evaluated in the spleen, liver, BM and dLN of the treated and infected animals, which were collected one and 15 days post-treatment, by a limiting dilution technique [64]. To this end, organs were macerated in a glass tissue grinder using sterile PBS, and tissue debris was removed by centrifugation at 150 ×*g*. Cells were centrifuged at 2000 ×*g* and pellets were resuspended in 1 mL of complete Schneider’s medium, when a log-fold serial dilution was performed in complete Schneider’s medium. Each sample was plated in triplicate and read 7 days after the beginning of the cultures at 24 °C. Results were expressed as the negative log of the titer (the dilution corresponding to the last positive well) adjusted per milligram of organ. Spleen parasitism was also evaluated by quantitative PCR (qPCR) [5, 20]. For this step, DNA was extracted using a commercial kit (Wizard Genomic DNA Purification Kit, Promega Corporation, Madison, WI, USA) and resuspended in milli-Q water. The following primers were used to amplify *L. infantum* kDNA: *Forward* (CCTATTTTACACCAACCCCCAGT) and *Reverse* (GGGTAGGGGCGTTCTGCGAAA), while the β-actin gene (*Forward*: CAGAGCAAGAGAGGTATCC; *Reverse*: TCATTGTAGAAGGTGTGGTGC) was used as a control. Standard curves were obtained from DNA extracted from 10^8^ parasites for kDNA and 10^8^ macrophages for β-actin, using the same technical conditions. Reactions were performed and analyzed in an ABI Prism 7500 Sequence Detection System (96 wells-plate; Applied Biosystems), using 2× SYBR™ Select Master Mix (Applied Biosystems), 2 mM of each primer and 25 ng/μL DNA. Samples were incubated at 95 °C for 10 min and submitted to 40 cycles of 95 °C for 15 s and 60 °C for 1 min. Fluorescence data were obtained at each time, and results were determined by interpolation from standard curves used in the same run, in duplicate, and expressed as the parasite number per total DNA.

### Cellular response

Mice spleens were collected one and 15 days after treatment, when they (5 × 10^6^ cells per mL) were incubated in 24-well plates (Nunc) cultured in DMEM plus 20% FBS and 20 mM L-glutamine at pH 7.4. Cells were unstimulated (medium) or stimulated with *L. infantum* SLA (50.0 μg/mL) for 48 h at 37 °C in 5% CO_2_. Levels of IFN-γ, IL-12, GM-CSF, IL-4, and IL-10 were measured in the culture supernatant by commercial kits obtained from BD Pharmingen^®^ (San Diego, CA, USA), according to the manufacturer’s instructions. Nitrite production was also evaluated in the cell supernatant by Griess reaction. In addition, in some culture wells, anti-CD4 (GK 1.5) and anti-CD8 (53-6.7) monoclonal antibodies (5.0 μg each; Pharmingen) were also added and processed as described above, aiming to evaluate the IFN-γ-producing T-cell profile in the miltefosine, b-AD or b-AD/Mic-treated mice groups. For this experiment, appropriate isotype-matched controls [rat IgG2a (R35-95) and rat IgG2b (95-1)] were also used. A flow cytometry assay was developed in the stimulated cultures to evaluate the IFN-γ and IL-10-producing CD4^+^ and CD8^+^ T cell frequency. Experiments were based on the cell relative flow cytometry size (forward laser scatter – FSC) and granularity (side laser scatter – SSC) graphs. After selection of the interest region R1 containing FSCLow and SSCLow phenotype cells, graphs of density plot distribution of CD4/FL1 or CD8/FL1 versus IFN-γ/FL2^+^, TNF-α/FL2^+^ and IL-10/FL2^+^ cells were constructed to determine the IFN-γ^+^, TNF-α and IL-10^+^-producing T cell frequency. Values were calculated by ratio between the CD4^+^ and CD8^+^ T-cell percentage in the SLA-stimulated versus unstimulated cultures, and they were expressed as indexes in the graphs [64].

### Antibody production

Levels of anti-*Leishmania* IgG1 and IgG2a isotype antibodies were measured in serum samples of the treated animals, which were obtained one and 15 days after treatment, as described in [25]. Briefly, SLA was added as an antigen to the plates, at a concentration of 1.0 μg per well, for 16 h at 4 °C. Free binding sites were then blocked with PBS and Tween 20 0.05% (PBS-T) plus 5% casein, for 1 h at 37 °C, when plates were washed five times with PBS-T and incubated with mouse sera (1:100 diluted in PBS-T), for 1 h at 37 °C. Afterwards, plates were washed five times and incubated with anti-mouse IgG1 and IgG2a horseradish-peroxidase conjugated antibodies (both 1:10,000 diluted in PBS-T), for 1 h at 37 °C. They were again washed and reactions were carried out using a solution composed of H_2_O_2_, ortho*-*phenylenediamine and citrate-phosphate buffer pH 5.0, for 30 min in the dark. Then, reactions were stopped by adding 2 N H_2_SO_4_, and OD values were read in a spectrophotometer, at 492 nm.

### *In vivo* toxicity

*In vivo* cytotoxicity was evaluated in serum samples of the treated and infected animals, one and 15 days post-treatment. For this evaluation, levels of aspartate aminotransferase (AST), alanine aminotransferase (ALT), and creatine kinase muscle brain fraction (CK-MB) were determined in the samples, using commercial kits (Labtest Diagnostica^®^, Belo Horizonte, Brazil) according to the manufacturer’s instructions.

### Statistical analysis

IC_50_, CC_50_ and RBC_50_ values were entered into Microsoft Excel (version 10.0) spreadsheets and calculated by dose-response curves, which were plotted in GraphPad Prism 5.03. Results were evaluated by the one-way analysis of variance (ANOVA), followed by Bonferroni’s post-test. Data were expressed as mean ± standard deviation of the groups. Two independent experiments presenting similar results were performed and differences were considered significant when *p* < 0.05.

## Results

### *In vitro* biological assays

**Figure 2:**
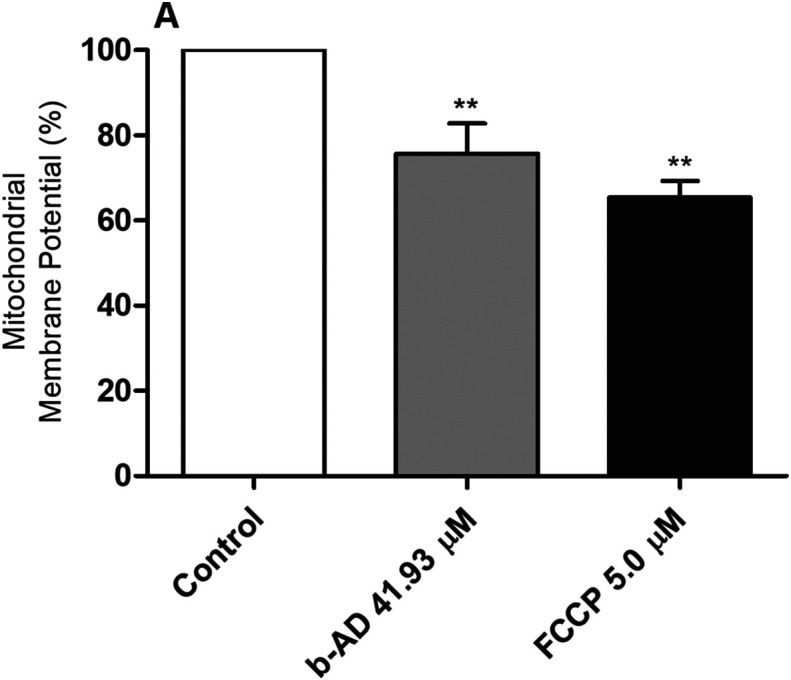
Evaluation of mitochondrial membrane potential. *Leishmania infantum* (10^7^ cells) were incubated alone (control) or in the presence of b-AD (41.93 μM, corresponding to two times the IC_50_ value) for 24 h at 25 °C. Parasites were then incubated with MitoTracker Red CM-H2XROS for 30 min and in the dark. Cells were washed twice with PBS and transferred to a black 96-well plate, when the fluorescence intensity was evaluated using a fluorometer. Promastigotes treated with carbonyl cyanide-4-(trifluoromethoxy)phenylhydrazone (FCCP; 5.0 μM) were used as a positive control, while those untreated were used as a negative control (control). Bars represent the mean plus standard deviation of the groups. (**) indicates a statistically significant difference as compared to the control (*p* < 0.0001).

**Figure 3:**
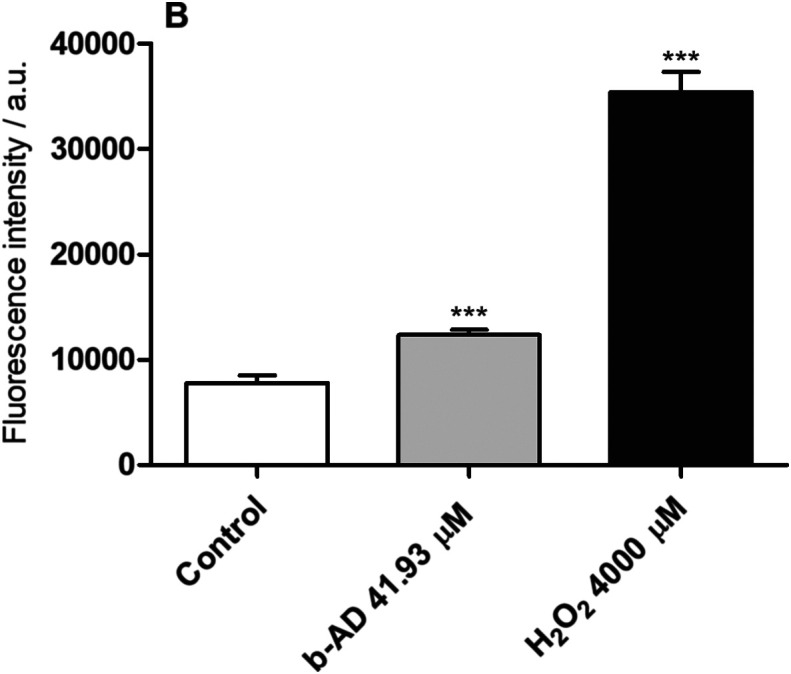
Production of reactive oxygen species. *Leishmania infantum* (10^7^ cells) were incubated alone (control) or in the presence of b-AD (41.93 μM) for 24 h at 25 °C. Parasites were then washed twice in PBS and incubated with 20 μM cell-permeant 2′,7′-dichlorodihydrofluorescein diacetate (H_2_DCFDA) for 30 min and in the dark. Fluorescence was measured in a fluorometer, with excitation and emission wavelengths of 485 and 528 nm, respectively. H_2_O_2_ (4000 μM; Sigma-Aldrich, USA)-treated parasites were used as a positive control, while untreated parasites were used as a negative control (control). Bars represent the mean plus standard deviation of the groups. (***) indicates a statistically significant difference as compared to the control (*p* < 0.0001).

The *in vitro* antileishmanial effect of the compounds was evaluated against *L. infantum* species, where IC_50_ values of 20.94 ± 2.60 and 0.11 ± 0.03 μM were obtained for b-AD and AmpB, respectively (Table 1). In turn, CC_50_ and RBC_50_ values determined for b-AD were 453.04 ± 23.29 and 334.95 ± 24.90 μM, respectively, and 0.86 ± 0.11 and 12.66 ± 1.52 μM for AmpB, respectively. The calculated selectivity indexes were 21.64 and 7.82 for b-AD and AmpB, respectively (Table 1). Treatment of infected macrophages reduced the infection percentage by 89.0% and 71.2%, when b-AD and AmpB were tested at 12.39 and 1.08 μM, respectively (Table 2). The inhibition of infection using pre-treated parasites was also evaluated, and results showed reductions in the infection percentage by 84.8% and 68.2%, when b-AD and AmpB were assayed at 12.39 and 1.08 μM, respectively (Table 3). Preliminary investigations of the effects of b-AD in *L. infantum* suggested that the compound caused alterations in parasite mitochondria, by altering their membrane potential (Fig. 2) and causing an increase in ROS production (Fig. 3).

**Table 1:** *In vitro* biological assays. *Leishmania infantum* logarithmic phase promastigotes were incubated with b-AD (0–61.96 μM) or AmpB (0–1.08 μM) for 48 h at 24 °C. Cell viability was analyzed by the MTT method and the 50% *Leishmania* inhibitory concentration (IC_50_) was calculated by applying sigmoidal regression of dose-response curve. Murine macrophages were also incubated with b-AD (0–123.92 μM) or AmpB (0–10.82 μM) for 48 h at 37 °C in 5% CO_2_, and the 50% cell inhibitory concentration (CC_50_) was also evaluated by a dose-response curve. The selectivity index (SI) was calculated by the ratio between the CC_50_ and IC_50_ values. The 50% human red blood cells inhibitory concentration (RBC_50_) was determined by incubating the 5% red cell suspension with b-AD (0–123.92 μM) or AmpB (0–10.82 μM) for 1 h at 37 °C in 5% CO_2_. The lysis percentage was evaluated spectrophotometrically, and the absence or presence of hemolysis were determined by replacing b-AD for PBS or distilled water, respectively. Results are shown as mean ± standard deviation of the groups.

Compound	IC_50_ (μM)	CC_50_ (μM)	SI	RBC_50_ (μM)
β-acetyl-digitoxin	20.94 ± 2.60	453.04 ± 23.29	21.64	334.95 ± 24.90
Amphotericin B	0.11 ± 0.03	0.86 ± 0.11	7.82	12.66 ± 1.52

**Table 2:** Evaluation of treatment of infected macrophages. Murine macrophages (5 × 10^5^ cells) were cultured in complete RPMI 1640 medium for 24 h at 37 °C in 5% CO_2_. After, cells were washed twice with PBS and *L. infantum* stationary promastigotes were added to the cultures at a ratio of 10 parasites per macrophage, for 48 h at 37 °C in 5% CO_2_. Free parasites were removed by washing with RPMI 1640 medium, and infected macrophages were treated with b-AD (0, 3.09, 6.19 and 12.39 μM) or AmpB (0, 0.27, 0.54 and 1.08 μM) for 48 h at 24 °C in 5% CO_2_. The percentage of infected cells, the infectiveness reduction, and the number of recovered amastigotes per cell were determined by counting 200 macrophages in triplicate. Results are shown as mean ± standard deviation of the groups.

Compound	Concentration (μM)	Percentage of infected macrophages after treatment	Infectiveness reduction (%)	Number of amastigotes per macrophage
β-acetyl-digitoxin	12.39	7.6 ± 1.2	89.0	0.1 ± 0
	6.19	14.3 ± 2.5	79.2	0.4 ± 0.2
	3.09	24.3 ± 3.3	64.6	1.2 ± 0.4
	0	68.7 ± 4.0	(–)	3.9 ± 0.5
Amphotericin B	1.08	19.8 ± 2.4	71.2	0.8 ± 0.4
	0.54	32.1 ± 3.8	53.3	1.3 ± 0.3
	0.27	42.5 ± 5.2	38.1	2.1 ± 0.4
	0	68.7 ± 4.0	(–)	3.9 ± 0.5

**Table 3:** Inhibition of infection of macrophages. The inhibition of infection of murine macrophages using pre-treated parasites was performed by incubating *L. infantum* stationary promastigotes (5 × 10^6^ cells) with b-AD (0, 3.09, 6.19 and 12.39 μM) or AmpB (0, 0.27, 0.54 and 1.08 μM) for 4 h at 24 °C. Parasites were then washed in RPMI 1640 and used to infect murine cells at a ratio of 10 parasites per macrophage, for 48 h at 37 °C in 5% CO_2_. The percentage of infected macrophages, the infectiveness reduction, and the number of amastigotes per treated macrophage were evaluated by counting 200 macrophages in triplicate. Results are shown as mean ± standard deviation of the groups.

Compound	Concentration (μM)	Infection percentage using pre-treated parasites	Infectiveness reduction (%)	Number of amastigotes per macrophage
β-acetyl-digitoxin	12.39	8.8 ± 2.0	84.8	0.2 ± 0.1
	6.19	13.3 ± 2.4	77.0	0.7 ± 0.3
	3.09	27.4 ± 4.0	52.6	1.6 ± 0.5
	0	57.8 ± 5.2	(–)	3.3 ± 0.6
Amphotericin B	1.08	18.4 ± 2.6	68.2	0.7 ± 0.3
	0.54	26.5 ± 2.7	54.2	1.2 ± 0.3
	0.27	38.7 ± 4.3	33.0	2.2 ± 0.4
	0	57.8 ± 5.2	(–)	3.3 ± 0.6

### Estimation of the parasite load

**Figure 4:**
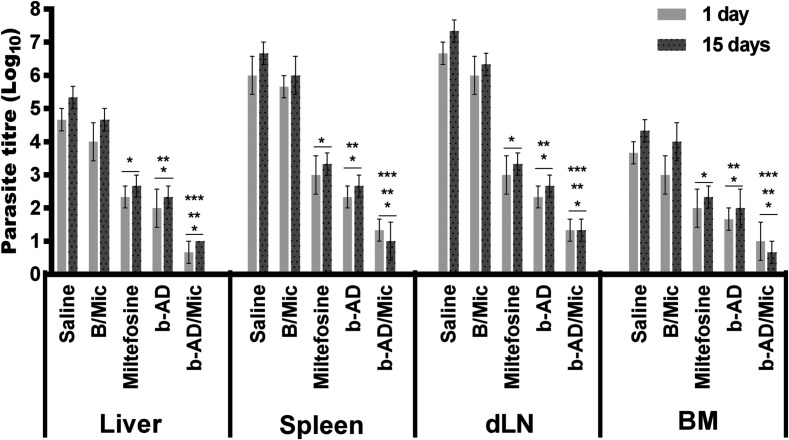
Organ parasitism evaluated in the treated animals by a limiting dilution technique. Mice (*n* = 12 per group) were infected with *L. infantum* and, 1 and 15 days after the distinct treatment schedules, they (*n* = 6 per group in each time) were euthanized and their livers, spleens, bone marrow (BM) and draining lymph nodes (dLNs) were collected to evaluate the parasite load, by a limiting dilution technique. Bars represent the mean ± standard deviation of the groups. (*) indicates a significant difference as compared to the saline and B/Mic groups (*p* < 0.05). (**) indicates a statistically significant difference as compared to the miltefosine group (*p* < 0.05). (***) indicates a statistically significant difference as compared to the b-AD group (*p* < 0.05).

**Figure 5:**
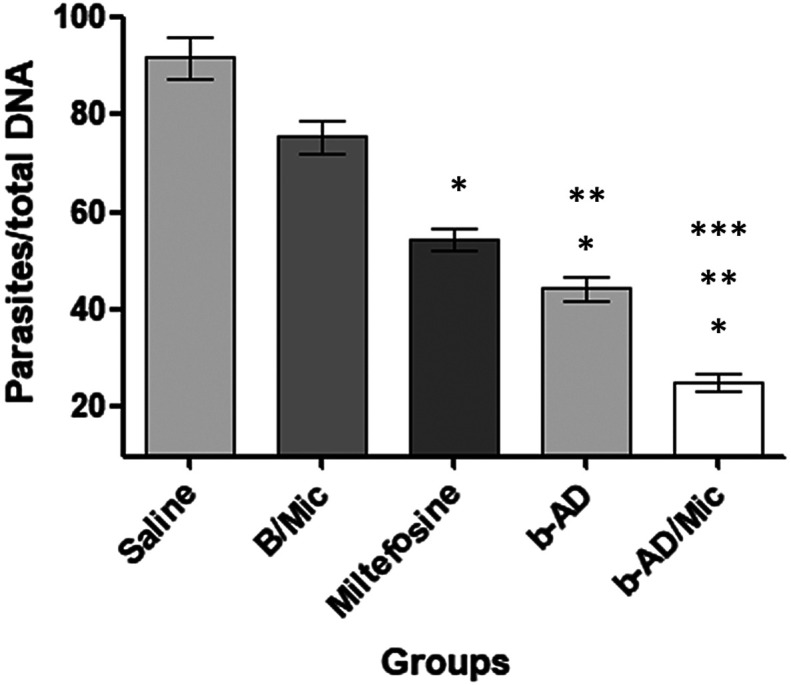
Splenic parasitism estimated by quantitative PCR. *Leishmania infantum*-infected mice (*n* = 6 per group) were submitted to the distinct treatment regimens and, 15 days post-therapy, they were euthanized and their spleens were collected to evaluate parasitism by qPCR. Results were expressed as the number of parasites per total DNA, and bars represent the mean ± standard deviation of the groups. (*) indicates a significant difference as compared to the saline and B/Mic groups (*p* < 0.05). (**) indicates a statistically significant difference as compared to the miltefosine group (*p* < 0.05). (***) indicates a statistically significant difference as compared to the b-AD group (*p* < 0.05).

Organ parasitism was evaluated one and 15 days post-treatment in liver, spleen, BM and dLNs of the treated animals. Results showed that the miltefosine, free b-AD or b-AD/Mic group mice presented significant reductions in the parasite load as compared to values encountered in the saline and B/Mic groups (Fig. 4). One day post-therapy, miltefosine, b-AD and b-AD/Mic group mice presented lower parasitism in their spleens (3.0, 3.7 and 4.6-log reductions, respectively), livers (2.3, 2.7 and 4.0-log reductions, respectively), dLNs (3.7, 4.3 and 5.3-log reductions, respectively), and BMs (1.7, 2.0 and 2.7-log reductions, respectively), when compared to the saline group. Fifteen days after treatment, reductions in the parasite load in the miltefosine, b-AD and b-AD/Mic groups were in the order of 3.3, 4.0 and 5.7-log reductions, respectively, in their spleens; of 2.7, 3.0 and 4.3-log reductions, respectively, in their livers; of 4.0, 4.7 and 6.0-log reductions, respectively, in their dLNs; and of 2.0, 2.3 and 3.7-log reductions, respectively, in their BM; when compared to the saline group. Comparison between the treated groups revealed that mice receiving b-AD/Mic showed the highest reductions in organ parasitism. The spleens of the animals were also used to determine the parasite load by qPCR technique, and results also showed that miltefosine, free b-AD or b-AD/Mic-treated mice presented significant reductions in splenic parasitism, when compared to values found in the control groups. Additionally, b-AD/Mic also induced the highest reductions in splenic parasitism as compared to the others (Fig. 5).

### Immune response in the treated animals

**Figure 6:**
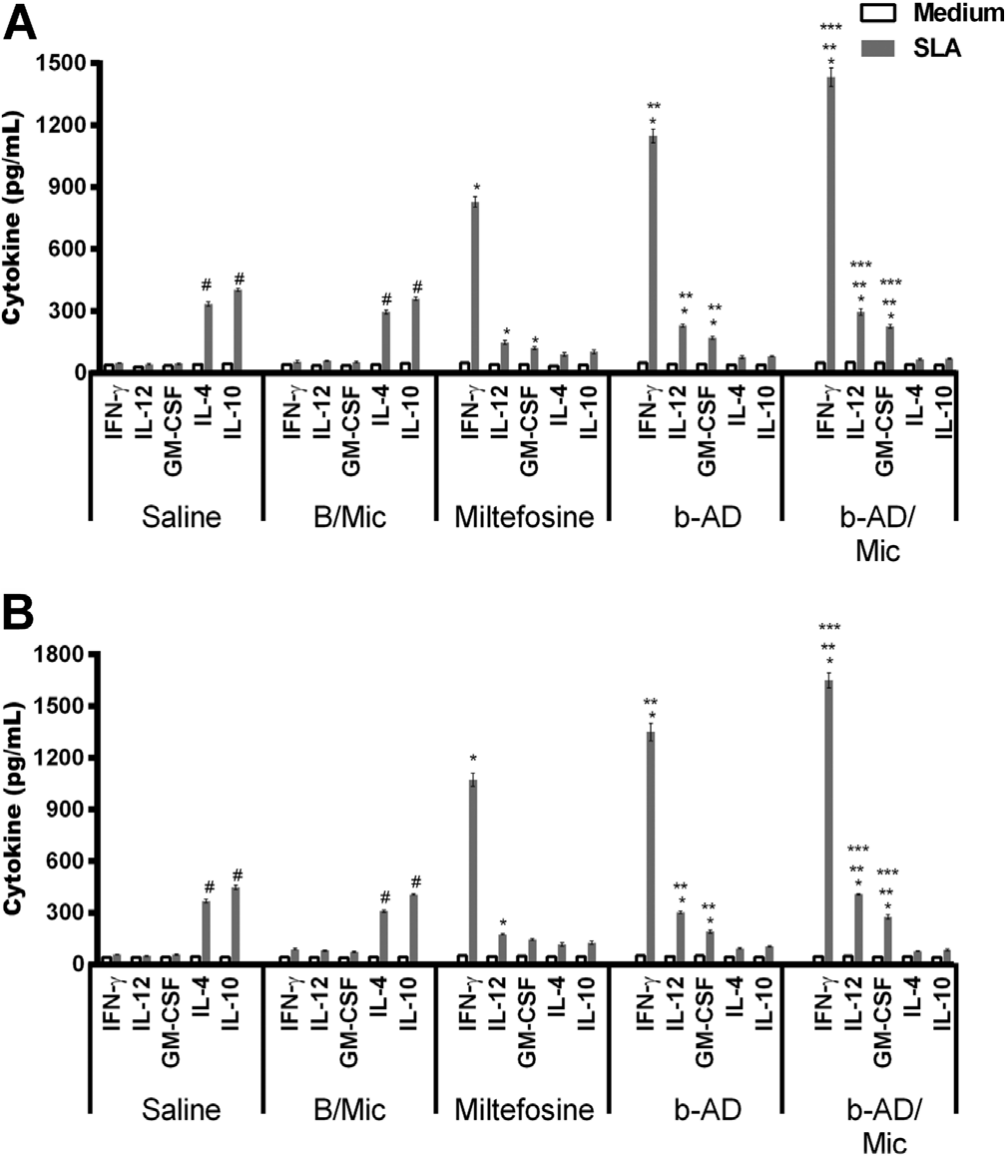
Cellular response generated in the treated animals. BALB/c mice (*n* = 12 per group) were infected with *L. infantum* promastigotes and submitted to distinct treatment schedules. 1 and 15 days post-therapy, they were euthanized and their spleens were collected and splenocytes were unstimulated (medium) or stimulated with *L. infantum* SLA (50.0 μg/mL), for 48 h at 37 °C in 5% CO_2_. Levels of IFN-γ, IL-4, IL-10, IL-12 and GM-CSF were quantified in the cell supernatant, one (A) and 15 (B) days post-treatment, by capture ELISA. Bars represent the mean ± standard deviation of the groups. (*) indicates a significant difference as compared to the saline and B/Mic groups (*p* < 0.05). (**) indicates a statistically significant difference as compared to the miltefosine group (*p* < 0.05). (***) indicates a statistically significant difference as compared to the b-AD group (*p* < 0.05). (#) indicates a statistically significant difference as compared to the miltefosine, b-AD and b-AD/Mic groups (*p* < 0.05).

**Figure 7:**
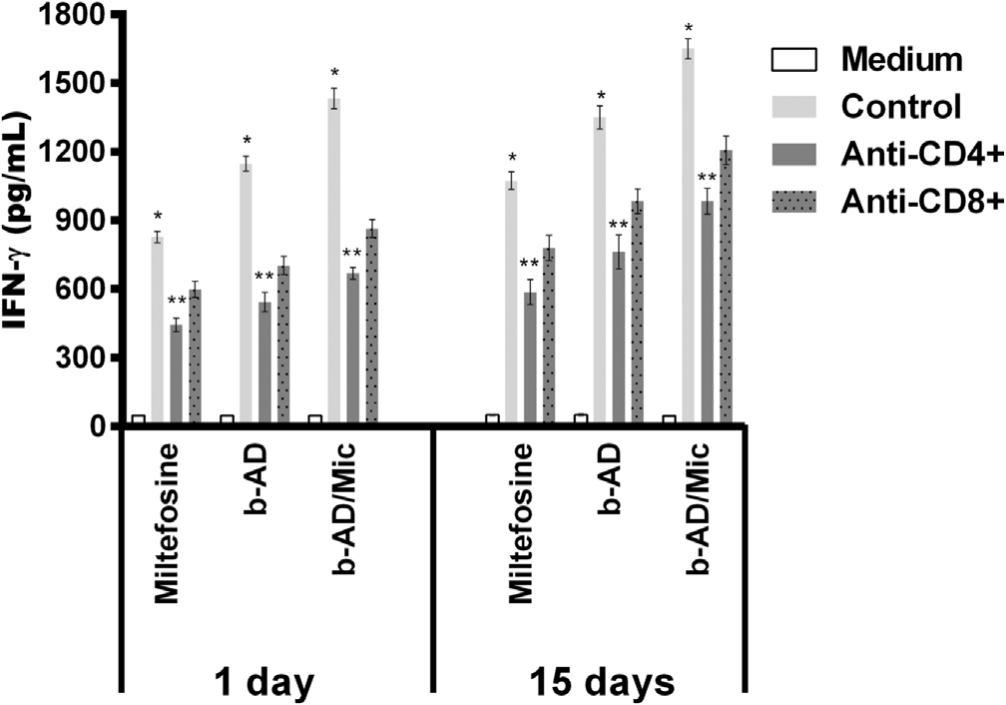
T-cell subtypes involved in the IFN-γ production. Splenocytes of mice treated with miltefosine, b-AD or b-AD/Mic (*n* = 6 per group) were cultured in DMEM (medium) or stimulated with SLA (50.0 μg/mL) in the presence of anti-CD4 or anti-CD8 antibody, for 48 h at 37 °C in 5% CO_2_. IFN-γ levels were quantified in the cell supernatant by a capture ELISA. Bars represent the mean ± standard deviation of the groups. (*) indicates a statistically significant difference as compared to the use of anti-CD4 or anti-CD8 antibody (*p* < 0.05). (**) indicates a statistically significant difference as compared to the use of anti-CD8 antibody (*p* < 0.05).

**Figure 8:**
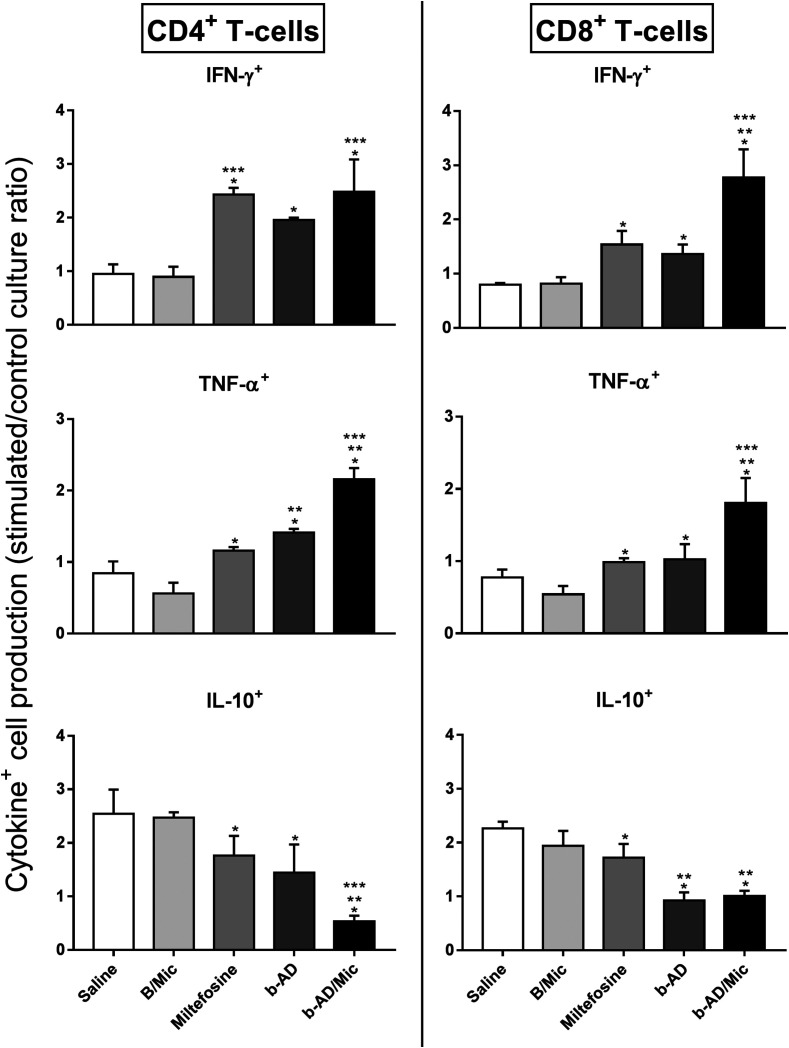
Investigation of intracytoplasmic cytokine-producing T cell frequency. Cytometry flow was performed to evaluate the IFN-γ, TNF-α and IL-10-producing T CD4^+^ and CD8^+^ cell frequency in splenocytes of mice treated, 15 days post-therapy. Results were calculated by the ratio between SLA-stimulated versus unstimulated cultures (SLA/CC ratio), and reported as cytokine indexes for CD4^+^ and CD8^+^ T cells. Bars represent the mean plus standard deviation of the groups. (*) indicates a significant difference as compared to the saline and B/Mic groups (*p* < 0.05). (**) indicates a statistically significant difference as compared to the miltefosine group (*p* < 0.05). (***) indicates a statistically significant difference as compared to the b-AD group (*p* < 0.05).

**Figure 9:**
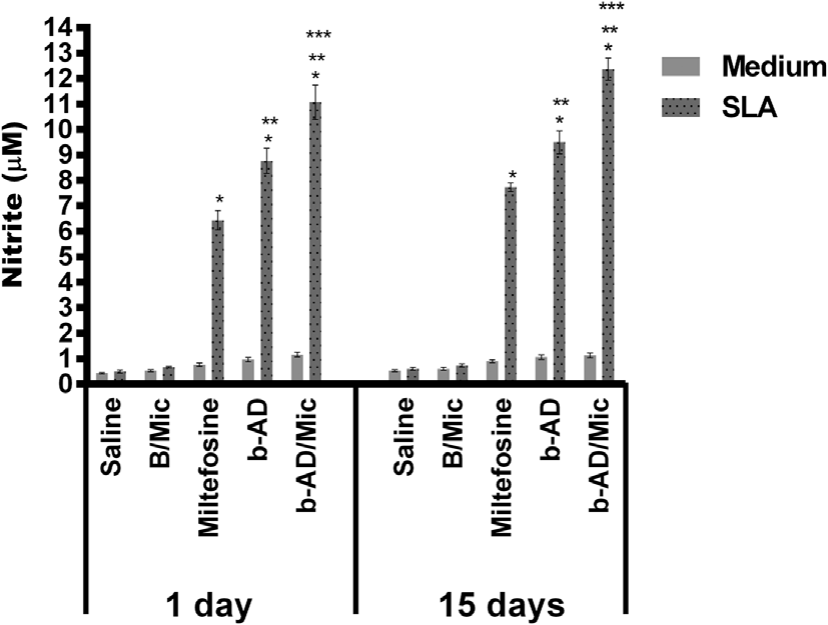
Nitrite production evaluated by Griess reaction. The cellular supernatants used to quantify cytokines in both periods of time after treatments were also used to evaluate SLA-specific nitrite production. Bars represent the mean ± standard deviation of the groups. (*) indicates a significant difference as compared to the saline and B/Mic groups (*p* < 0.05). (**) indicates a statistically significant difference as compared to the miltefosine group (*p* < 0.05). (***) indicates a statistically significant difference as compared to the b-AD group (*p* < 0.05). (#) indicates a statistically significant difference as compared to the miltefosine, b-AD and b-AD/Mic groups (*p* < 0.05).

**Figure 10:**
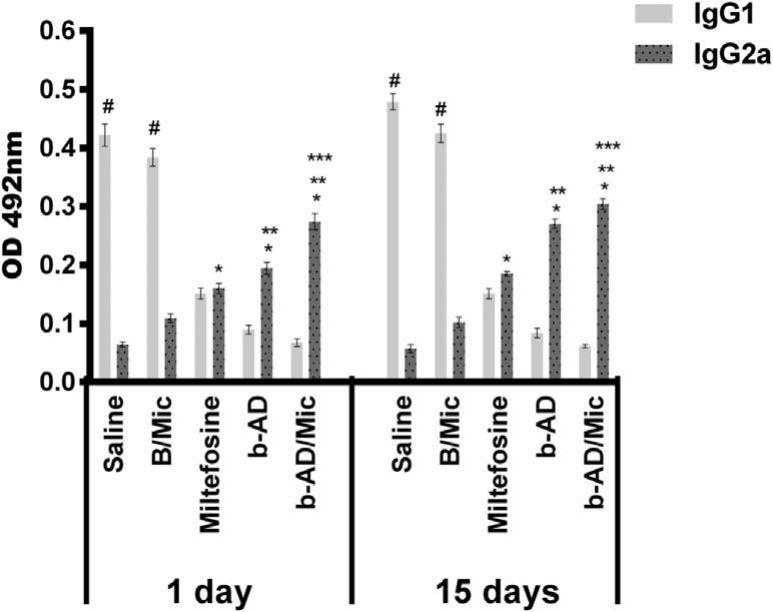
Antibody response produced in the treated animals. Serum samples were collected of mice infected and later treated, 1 and 15 days post-therapy, and levels of anti-parasite IgG1 and IgG2a isotypes were investigated by ELISA. Bars represent the mean ± standard deviation of the groups. (*) indicates a significant difference as compared to the saline and B/Mic groups (*p* < 0.05). (**) indicates a statistically significant difference as compared to the miltefosine group (*p* < 0.05). (***) indicates a statistically significant difference as compared to the b-AD group (*p* < 0.05). (#) indicates a statistically significant difference as compared to the miltefosine, b-AD and b-AD/Mic groups (*p* < 0.05).

Anti-parasite Th1 and Th2-type cytokines were evaluated in the cell supernatant of splenocyte cultures of the treated animals. Results showed that spleen cells of miltefosine, b-AD or b-AD/Mic groups mice produced significantly higher levels of IFN-γ, IL-12 and GM-CSF, as well as low levels of IL-4 and IL-10, when compared to data found in the saline and B/Mic groups, when both periods of time were evaluated (Fig. 6). Aiming to investigate the T cell subtype responsible by IFN-γ production in the treated and infected animals, anti-CD4 and anti-CD8 monoclonal antibodies were added to the *in vitro* cultures, and results showed that both antibody subtypes reduced production of this cytokine in significant levels. This suggests that CD4^+^ and CD8^+^ T cells were important for immunological response against *L. infantum* infection (Fig. 7). A flow cytometry assay also showed that miltefosine, b-AD and b-AD/Mic-treated mice presented higher IFN-γ and TNF-α-producing T-cell subtype frequency as compared to data obtained in the saline and B/Mic groups, which showed higher IL-10-producing CD4^+^ and CD8^+^ T-cell levels (Fig. 8). Between the therapeutics, b-AD/Mic induced higher presence of IFN-γ and TNF-α-producing CD4^+^ and CD8^+^ T cells. Nitrite production was evaluated by Griess reaction, and results showed that miltefosine, b-AD or b-AD/Mic group mice produced higher levels of this cell activation marker, when compared to values found in the saline and B/Mic groups (Fig. 9). The evaluation of humoral response also showed that miltefosine, b-AD or b-AD/Mic group mice produced significantly higher levels of anti-*Leishmania* IgG2a antibody, as compared to IgG1 levels. Otherwise, saline and B/Mic group mice produced higher anti-parasite IgG1 isotype levels than IgG2a isotype, in both periods of time post-treatment (Fig. 10). The treatment using b-AD/Mic induced the highest levels of anti-parasite IgG2a antibodies as compared to the other groups.

### *In vivo* toxicity

**Figure 11:**
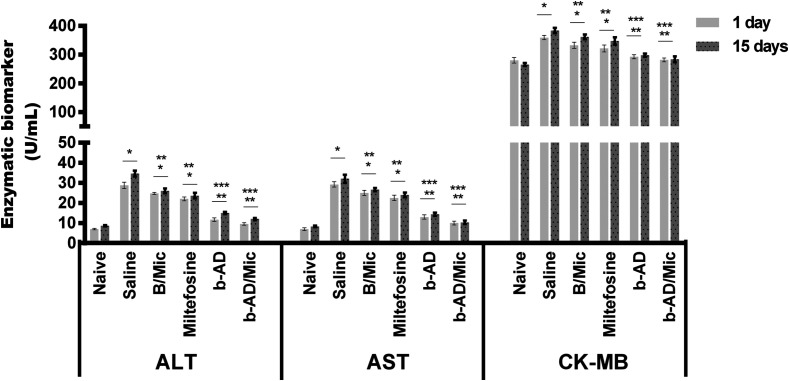
*In vivo* cytotoxicity. To investigate the *in vivo* toxicity of the therapeutics, levels of alanine aminotransferase (ALT), aspartate aminotransferase (AST), and creatine kinase muscle brain fraction (CK-MB) enzymes were quantified in serum samples of mice infected and treated, 1 and 15 days post-therapy. Samples of naive (non-infected and non-treated) mice were used as control. Bars represent the mean ± standard deviation of the groups. (*) indicates a significant difference as compared to the saline and B/Mic groups (*p* < 0.05). (**) indicates a statistically significant difference as compared to the miltefosine group (*p* < 0.05). (***) indicates a statistically significant difference as compared to the b-AD group (*p* < 0.05). (#) indicates a statistically significant difference as compared to the miltefosine, b-AD and b-AD/Mic groups (*p* < 0.05).

Hepatic and cardiac toxicity was evaluated in the treated and infected animals. Results showed higher levels of ALT, AST and CK-MB enzymes in the saline and B/Mic group mice, suggesting that organic changes could occur in these animals caused by infection and/or treatment. On the other hand, the miltefosine, b-AD and b-AD/Mic group mice produced lower levels of these enzymatic markers, with animals treated with b-AD/Mic being those presenting the lowest levels of AST, ALT and CK-MB (Fig. 11).

## Discussion

Drugs currently used for VL treatment are toxic, costly, have long treatment times to achieve a therapeutic effect and/or variable efficacy [61]. In this context, novel antileishmanial agents are needed and plant products may represent a valid strategy for drug development [53], especially compounds or derivatives in clinical use, such as cardenolide derivatives. Cardiac glycosides such as digoxin are used to heart diseases, and several compounds of this class have been described to induce a plethora of biological responses, including anti-inflammatory and antiprotozoal activities [14, 29, 68]. Following the rationale to identify novel antileishmanial targets, in the present work, a cardenolide derivative called β-acetyl-digitoxin, which was obtained from *Digitalis lanata* leaves, was evaluated regarding its *in vitro* and *in vivo* activity against *L. infantum*. The obtained results indicated the effectiveness of b-AD against the parasites, especially when it was tested incorporated in Poloxamer 407-based polymeric micelles. This composition was more efficient in reducing the parasite load in the treated and infected animals, and also in inducing a more polarized and specific antileishmanial Th1-type response.

The occurrence of active VL depends on the activation of T cell subsets, being Th1-type cytokines, such as IFN-γ, GM-CSF and IL-12, among others related to the protective response, while IL-4, IL-5, IL-6, IL-10, among others related to the development of the disease [16, 18, 31, 42]. Usually, protective immunity depends on the induction of specific Th1-type response, which activates macrophages to kill intracellular parasites through nitric oxide-mediated mechanism [30]. In this study, considering the analysis of T-cell populations and cytokine production, both CD4^+^ and CD8^+^ T subtypes were shown to be the origin of IFN-γ in mice that were treated with miltefosine, b-AD or b-AD/Mic. The Th1-type profile was related to parasitism control in distinct organs of the animals, when the parasite load was evaluated through a limiting dilution technique and qPCR. On the other hand, control group mice showed higher parasitism in these systemic organs, as well as the development of Th2-type immune response. Parasitological and immunological evaluations performed 1 and 15 days post-treatment indicated the maintenance of the positive therapeutic response in the treated animals, suggesting possible long-term efficacy of b-AD against *L. infantum*.

In our study, b-AD showed higher therapeutic efficacy when it was incorporated in Poloxamer 407 (Pluronic^®^ F127)-based polymeric micelles. The use of micelles as delivery vehicles for antileishmanial molecules has presented good therapeutic efficacy against *Leishmania* spp. [20, 34, 37]. In one study, AmpB-containing polymeric micelles were tested against *L. donovani*, and results showed that the composition was 100 times more active against parasites as compared to the use of free AmpB [23]. In another study, AmpB-containing and chitosan-coated Poloxamer 407-based micelles were prepared and the composition was tested against *Leishmania*, with results showing low toxicity of formulation in mammalian cells, higher uptake by host macrophages and higher efficacy against infection by the parasites [54].

Our group also used polymeric micelles as delivery systems for antileishmanial candidates. In a study, AmpB-containing Poloxamer P407 micelles were prepared and tested against *L. amazonensis*. Results showed that the composition induced significant reductions in the parasite load in infected BALB/c mice, as compared to the other groups. Th1-type immunity developed in these treated animals, which was associated with the therapeutic response against infection [37]. In another study, clioquinol-containing Pluronic^®^ F127 polymeric micelles (ICHQ/Mic) were developed and tested against *L. amazonensis.* Results showed that treated mice developed a Th1-type immune response, which was associated with significant reductions in parasitism in livers, spleens, BMs and dLNs of the animals, when compared to data found in the control groups [64].

Cardenolides have been used to treat congestive heart failure and arrhythmias [1, 11, 32, 52]. In the present study, preliminary data suggest that the mechanism of action of our cardenolide derivative in *L. infantum* involved the parasite mitochondria causing cell death, since b-AD altered mitochondrial membrane potential and stimulated ROS production by the parasites. In fact, the presence of high levels of ROS can cause increased levels of lipid peroxidation and reduction in membrane fluidity, leading to the loss of cell viability [55, 58]. Mitochondria have been considered a target when distinct antileishmanial agents were tested [7, 28, 35, 57, 63]; however, contrarily to what was observed in mammalian cells, where this organelle is abundant in their content, *Leishmania* spp. and other trypanosomatids present only one mitochondrion, which exhibits functional and morphological differences, such as the presence of its own genetic material, peculiarities in the functionality of the electron transport chain, and existence of non-canonical antioxidant machinery [2, 24, 40]. In this context, and based on the data presented here, b-AD was able to induce a stress environment in the parasites, which contributed to *Leishmania* death. Additionally, and due to the low toxicity found in murine and human cells, one could speculate that this compound will not be toxic to treat human VL, mainly when administered at low doses and/or incorporated in delivery systems such as polymeric micelles.

Miltefosine is used as an oral drug for the treatment against VL; however, its effectiveness has been variable between different populations [51, 60]. In addition, due to its long half-life and long treatment duration, parasite resistance against this drug has also been documented [45, 47]. Here, miltefosine induced significant reductions in parasitism in the treated animals, as compared to values found in the controls; however, data obtained in b-AD/Mic-treated mice, showing more significant reductions in the parasite load, suggest that the micellar composition was more effective in reducing organ parasitism in treated and infected animals. Taken together, these results suggest that b-AD/Mic could be considered effective against *L. infantum* infection in our experimental mammalian model.

Infection by *L. infantum* usually causes primary parasitism in the liver of the animals that tends to decrease, while splenic parasitism tends to increase when the infection becomes chronic [12, 43]. In this context, animal spleens could be considered a marker of chronic infection [22, 36]. In our study, parasitism in this organ was evaluated by two distinct techniques and results showed that when both assays were performed, lower splenic parasitism was found in b-AD/Mic-treated mice, as compared to the other groups, including the treatment using miltefosine. This fact could be considered relevant and therapeutic schemes testing the association of this cardenolide derivative with other antileishmanial drugs could be considered. The purpose would be to reduce the side effects, decrease parasite resistance, and allow the prescription of lower drug doses to achieve satisfactory therapeutic results [30, 48, 59, 65].

Limitations of the study include the absence of other therapeutic schedules employing lower numbers of doses and other antileishmanial agents, such as amphotericin B, as well as the absence of parasitological and immunological evaluations performed over longer periods of time post-treatment, aiming to verify possible long-term therapeutic efficacy induced by b-AD/Mic. In addition, the absence of evaluation of levels of urea and creatinine in the serum samples of treated and infected animals could be considered a limitation of the work, since antileishmanial agents, such as amphotericin B [38, 50], cardenolide derivatives [9, 17], among others, can cause renal toxicity, mainly if administered in high and/or inappropriately doses [9]. In this context, additional studies are needed, aiming to solve such questions. However, preliminary data presented here, describing the parasitological and immunological therapeutic response found in mice treated with b-AD/Mic, as well as the low cardiac and hepatic toxicity, suggest that this compound could be considered in future studies as a therapeutic agent against VL.

## Conflict of interest

The authors confirm that they have no conflicts of interest in relation to this work.

## Notes

**Cite this article as**: Freitas CS, Lage DP, Oliveira-da-Silva JA, Costa RR, Mendonça DVC, Martins VT, Reis TAR, Antinarelli LMR, Machado AS, Tavares GS, Ramos FF, Brito RCF, Ludolf F, Chávez-Fumagalli MA, Roatt BM, Ramos GS, Munkert J, Ottoni FM, Campana PRV, Duarte MC, Gonçalves DU, Coimbra ES, Braga FC, Pádua RM & Coelho EAF. 2021. *In vitro* and *in vivo* antileishmanial activity of β-acetyl-digitoxin, a cardenolide of *Digitalis lanata* potentially useful to treat visceral leishmaniasis. Parasite **28**, 38.
